# Supplementary material for: Joint optimization of land carbon uptake and albedo can help achieve moderate instantaneous and long-term cooling effects
Source: Commun Earth Environ. 2023 Aug 25;4(1):298. doi: 10.1038/s43247-023-00958-4 (PMC11041785; doi:10.1038/s43247-023-00958-4)
Supplement: Supplementary file 1 — Supplementary Information [file 43247_2023_958_MOESM1_ESM.pdf]

Supplementary Information for

Joint optimization of land carbon uptake and albedo can help achieve moderate instantaneous and long-term cooling effects

Supplementary Methods 1: Measurement sites. Supplementary Figure 1 shows the global distribution of measurement sites with the required CO_2 and shortwave radiation flux measurements and Supplementary Figure 2 their distribution in precipitation-temperature space. In terms of the IGBP land cover classification, the 176 sites include 40 evergreen needleleaf forest ENF, 11 evergreen broadleaf forest EBF, 0 deciduous needleleaf forest, 25 deciduous broadleaf forest DBF, 8 mixed forests MF, 2 closed shrublands CSH, 5 open shrublands OSH, 7 woody savannas WSA, 9 savannas SAV, 32 grasslands GRA, 16 permanent wetlands WET, 18 croplands CRO, 0 urban, 0 mosaic, 3 snow and ice SNO, 0 barren, and 0 water bodies.

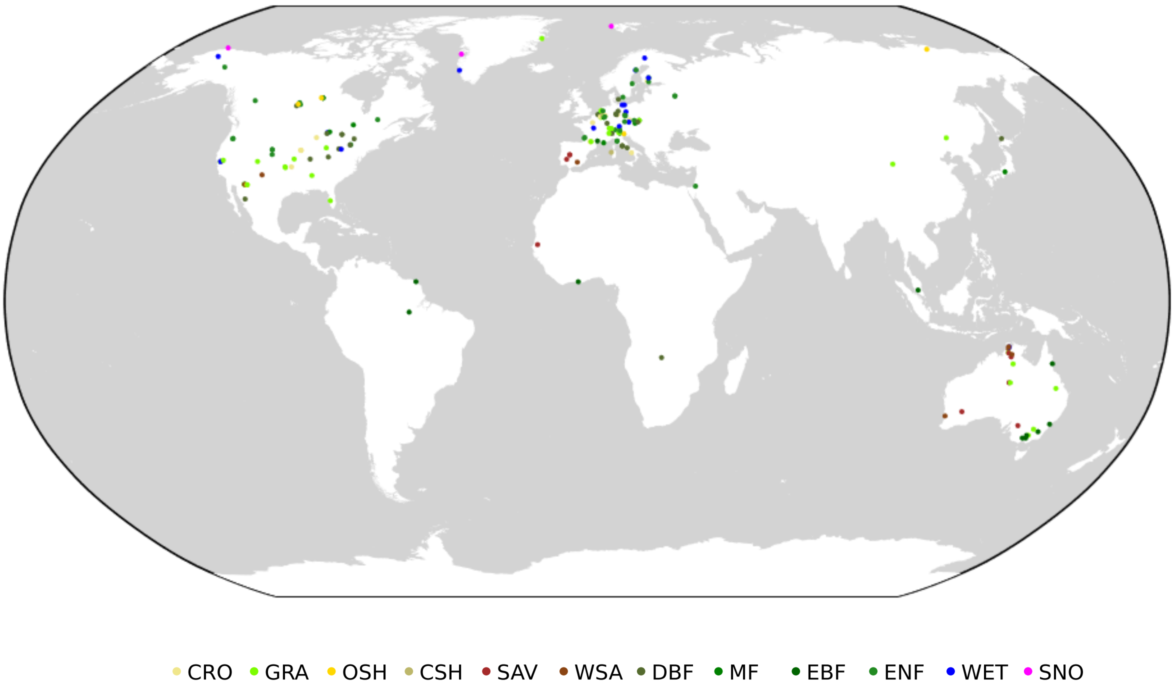

Supplementary Figure 1: Global distribution of used FLUXNET sites with sufficient coverage of the study core variables. Colours indicate IGBP land cover type. Mapping and background: basemap [1].

Supplementary Methods 2: Imputation of missing radiation values and snow detection. Time series of station measurements exhibit data gaps for reasons such as power failures, malfunctions, and, in case of turbulent fluxes, also unfavourable meteorological conditions. If not distributed randomly with respect to season, daytime and weather, such gaps might introduce systematic errors into annual or multi-annual averages. For the majority of variables used in this study, well-proven imputation methods were already included in the data product [2].

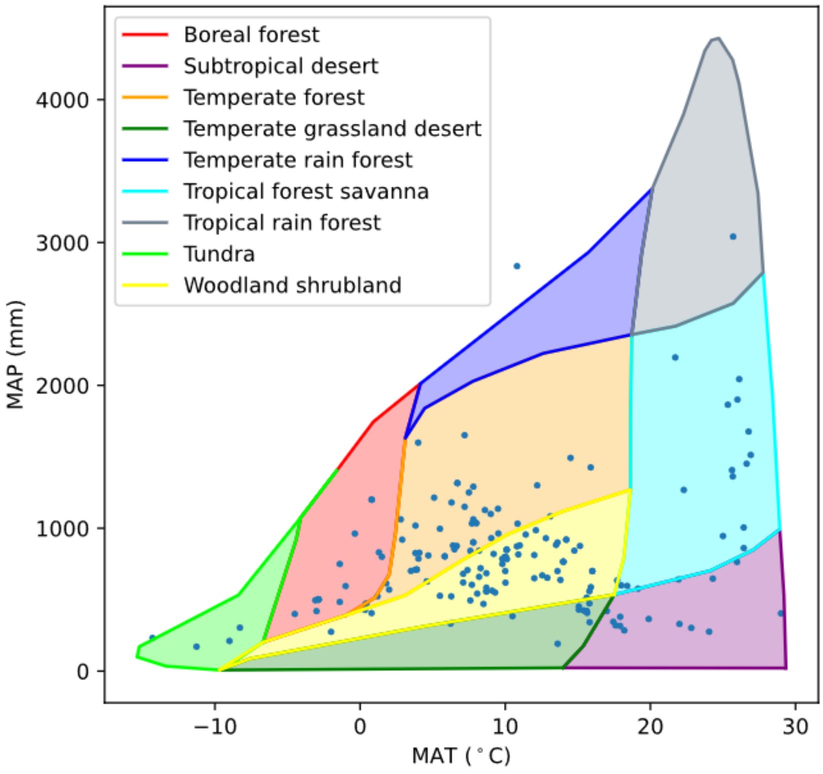

Supplementary Figure 2: Mean annual precipitation (MAP) and temperature (MAT) of the sites overlaid on the biome classification according to Whittaker [3,4,5]. Except for tropical (one site) and temperate (one outlier) rain forests, all biomes are represented by three or more sites.

For SW_{out}, we applied an adaptive window imputation method similar as used in the past for actual and potential evapotranspiration [6-8]. Ignoring smaller dependencies on insolation angle, SW_{out} and SW_{in} are proportional, with the proportionality constant α_s approximately constant over several days. After a time period not known a priori, the constant may change either gradually (e.g. due to phenology) or abruptly, in particular due to snowfall. Therefore, a window of a minimum length of at least 336 data points (corresponding to 7 days in case of half-hourly, gapless data) was gradually increased in steps of 48 records (1 day). For each increment, a tentative α_s was computed as the ratio of the window's mean SW_{out} to SW_{in}, and the mean square error of predicted ($\alpha_s SW_{in}$) vs. measured SW_{out} was determined. Iteration was stopped and the next window started upon the first local minimum of this error, but no gaps longer than 2976 records (62 days) were filled and not extrapolated at the start or end of a site's time series for more than 1920 records (40 days). The median, average and maximum percentage of filled gaps per site was 5.7%, 8.1% and 48.1%, respectively. A cross-validation with an additional, synthetic data gap per site of random, uniformly distributed length up to the abovementioned maximum allowed gap length revealed an R^2 of 0.87 and regression slope of 0.92 between the removed SW_{out} values and their inferred imputation values.

Each final window was flagged if its albedo and temperature data indicated the presence of a snow cover, and each site with any such time window in its time series was flagged as

snow-affected. For the detection of snow-affected time windows, all available final windows were first partitioned into a group with either minimum half-hourly air temperature below 1°C and mean air temperature below 15°C (chance for snow), or at least one of both values above the limit (snow-free). The median (MED) of the latter was used as a first order approximation of the site's albedo during snow-free periods and its median absolute deviation (MAD) a measure of its variability. The maximum albedo (MAX) during the former (chance for snow) situations was considered the most likely typical albedo under full snow cover and its difference towards MED (DIF) a measure of the site-specific albedo difference between snow-covered and snow-free situations. A window was considered snow-affected if its albedo was larger than MED + TOL, with the tolerance TOL = min(0.25*DIF, 10*MAD). For some (N=50) sites, estimates of the mean annual number of days with snow cover provided by the station operators was part of the FLUXNET2015 and ICOS dataset. These were used as an independent reference to measure the efficiency of the snow cover detection algorithm. As can be seen in Supplementary Figure 3, the agreement between both sources was fair given the inherent limitations of both methods. If anything, our automated algorithm tends to overestimate snow cover duration at sites with no or very rare snow events. Consequently, the sites flagged as snow-free in Fig. 1 a of the main text are highly reliably so, whereas the group of snow-affected sites may include false positives.

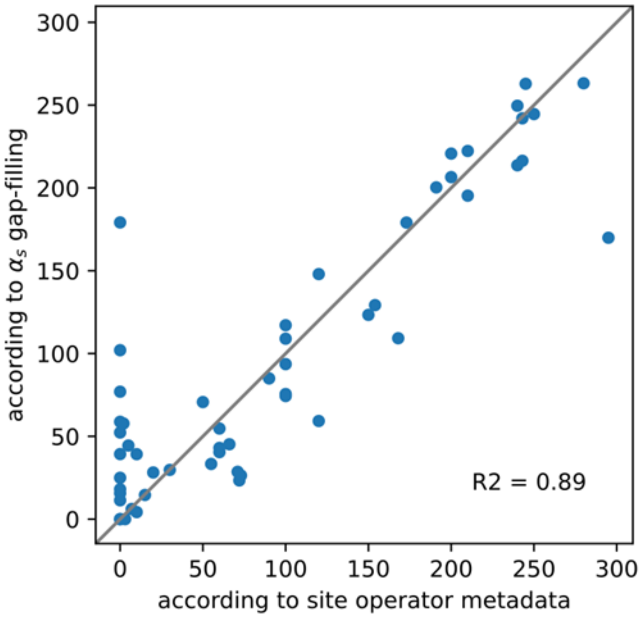

Supplementary Figure 3: **Comparison of average snow cover days per year according to a site operator estimate provided with the FLUXNET metadata (x axis) and according to our albedo gap-filling algorithm**. Grey: 1:1 line.

Supplementary Methods 3: Surface albedo effect at the top of the atmosphere. Modifications to the radiation budget following a change in albedo can be described by

$$\Delta R = (\mathrm{SW_{in}} + \Delta \mathrm{SW}_{in})(\alpha + \Delta\alpha) - \mathrm{SW_{in}}\alpha + \varepsilon, \qquad \text{(S1)}$$

where $\mathrm{SW_{in}}$ and α are the incoming short-wave radiation and albedo before modification, $\Delta\alpha$ the direct modification of albedo and $\Delta\mathrm{SW_{in}}$ any possible modification of $\mathrm{SW_{in}}$ caused by it, e.g. via changed latent heat flux and cloudiness. The residual ε summarizes any effect on the radiation budget that cannot be described by the former variables, e.g. changes in long-wave radiation indirectly caused by the albedo change via a modified temperature. While this way to separate direct ($\mathrm{SW_{in}}\,\Delta\alpha$) from indirect ($\varepsilon$ and products of $\Delta\mathrm{SW_{in}}$) effects cannot be translated exactly into the concept of instantaneous vs. effective radiative forcing (ERF [9,10]), the direct effect is part of the instantaneous radiative forcing (IRF) and the indirect effects overlap with the "rapid adjustments" (radiation-relevant changes to e.g. the atmosphere's thermal structure, clouds, or humidity [11]), which distinguish ERF from IRF and often cannot be detected in observations [9]. The magnitude of indirect effects associated with ε and $\Delta\mathrm{SW_{in}}$ depends on whether each of α, $\mathrm{SW_{in}}$ and R are defined at the surface or the top-of-atmosphere (TOA). If all quantities are consistently defined at TOA (i.e., α and its Δ correspond to a known combined albedo of surface and cloud cover), $\Delta\mathrm{SW_{in}}$ is zero and ε is only required to accommodate changes in long-wave radiation, which will result from the albedo modification via a change in surface and atmosphere temperature. Consequently (S1) simplifies to

$$\Delta R = \mathrm{SW_{in}}\Delta\alpha + \varepsilon, \qquad \text{(S2)}$$

with $\varepsilon << \mathrm{SW_{in}}\,\Delta\alpha$. The same applies if all quantities are defined at the surface and the modified surface area is too small to considerably change cloud coverage, and thus $\mathrm{SW_{in}}$ at the surface. Larger importance of ε arises if α is given at the surface while $\mathrm{SW_{in}}$ is only known, and R wanted, at the TOA. In this case ε includes the effect of cloud cover [12] on the difference between surface and TOA albedo.

In our case, α, $\Delta\alpha$ and $\mathrm{SW_{in}}$ are given at the surface, but for better comparability with CO_2-based radiative forcings ΔR is wanted at TOA, and $\Delta\mathrm{SW_{in}}$ and ε are unknown. The effect of a constant cloud cover can in this case be shown to mathematically cancel out, but atmospheric absorption, a modified partitioning between sensible and latent heat flux and resulting changes in long-wave radiation and cloud cover can still cause non-negligible $\Delta\mathrm{SW_{in}}$ and ε. An alternative to neglect or explicitly estimate each of these effects is the use of model-based radiation kernels. The kernel-based estimate of the surface albedo effect on ΔR at TOA at a particular surface location and month is given by [13]

$$\Delta R \approx k\Delta\alpha_{s,} \qquad \text{(S3)}$$

where the kernel k is derived by comparing the TOA radiation budget of a reference run of a gridded climate model to one where surface albedo was perturbed once for each grid point. The kernel has the same unit, approximate magnitude and spatiotemporal variation as $\mathrm{SW_{in}}$. Unlike measured $\mathrm{SW_{in}}$ at the surface it does not require a correction for static atmospheric absorption, and unlike SW_{in} at the TOA it does not require a correction for static surface albedo masking by clouds. However, the methodology to derive kernels does not include the atmospheric adjustments in which ERF differs from IRF. With presently available model outcomes, we can only present a rough estimate of these additional

effects. Assuming that a comparison of ERF and IRF of land-use change across 14 CMIP6 models [14] is dominated by albedo and CO_2 sequestration changes, the inter-model mean relative difference of - 36 % (Table 7 in [14], ERF less important than IRF) can serve as a rough indicator of a possible overestimation in our results. It should be noted, however, that the individual models contributing to this study saw adjustments of both positive and negative signs, with a standard deviation between models (0.08 W m^{-2}) larger than the inter-model mean adjustment (0.05 W m^{-2}).

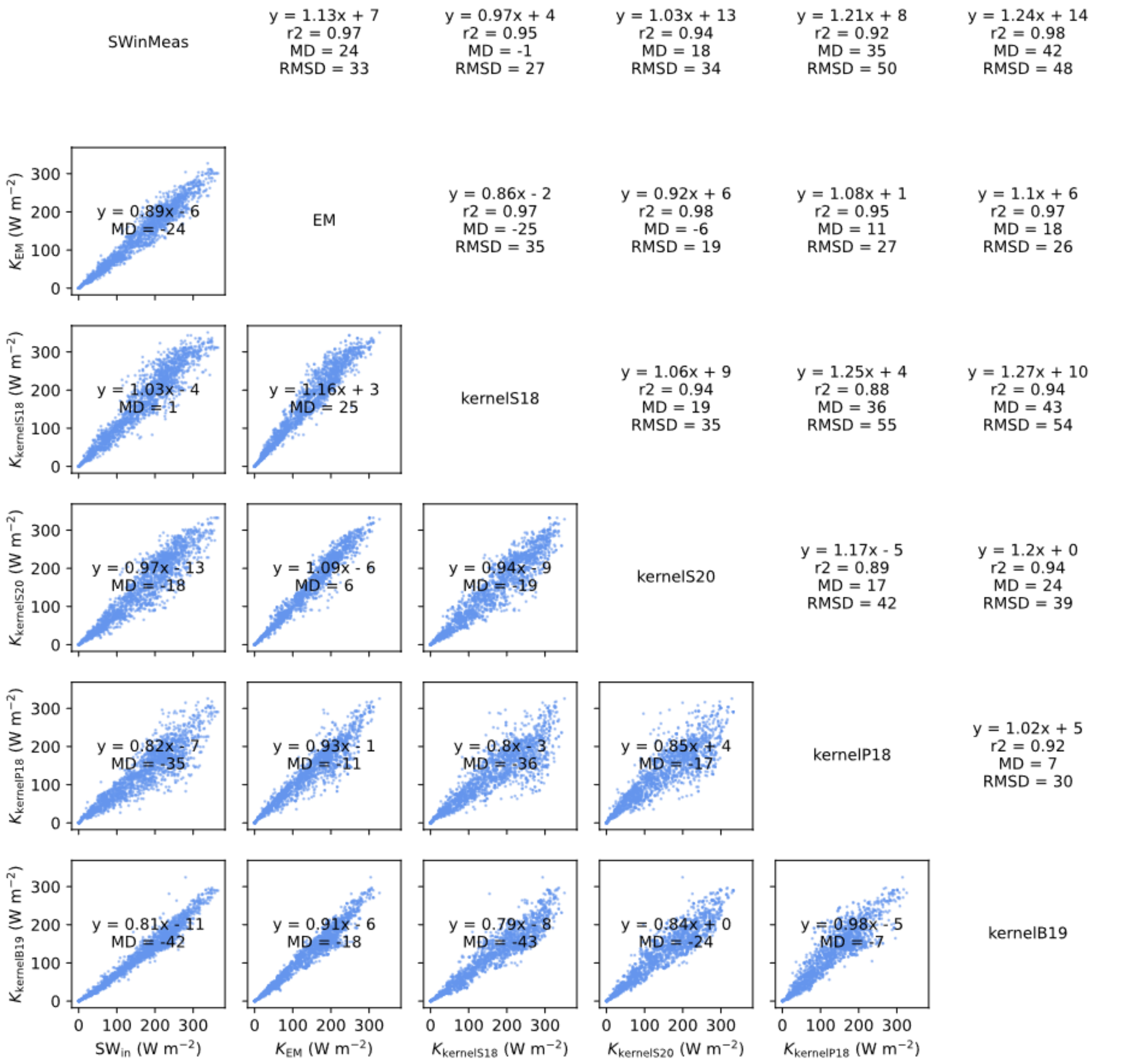

Supplementary Figure 4: Comparison between measured SW_{in} at the surface, surface albedo kernels from four different datasets [11,13-15] at the corresponding geographical coordinates, and their ensemble mean. Each data point corresponds to a climatological site-month, i.e. in case of multiple years of available measurements or modelling results, they were averaged across years but separately for each site and month. r2: coefficient of determination (squared Pearson correlation coefficient), MD: mean difference, RMSD: Root mean square difference. All regression-like empirical linear equations are based on the reduced major axis [16] technique, a subtype of what is sometimes referred to as model II regression in which the x and y variable have unknown errors and are interchangeable such that equation x(y) is the inverse of y(x).

We compared surface albedo kernels from four recently published datasets [11,13-15], their ensemble mean, and observed SW_{in} from our dataset to each other (Supplementary Figure 4). Per model-based dataset, kernels range from slightly larger than SW_{in} (S18 [11], factor 1.03) to almost 20% lower (B19 [13]). The pairwise coefficient of determination (r^2) between SW_{in} and each kernel dataset is > 0.9, but not all pairwise r^2 between the model datasets. The ensemble mean (EM) of all model-based datasets is highly correlated to SW_{in} and 11% lower than it, which is plausible given that atmospheric absorption should slightly reduce the TOA effect of surface albedo changes compared to their effect at the surface. In the main article, we solely use model-based kernel datasets to estimate the climate effect of albedo changes (see Supplementary Methods 5). For future studies without direct usage of model-based kernels, however, it is noteworthy that using measured SW_{in} at the surface with a correction factor (1.03 for the strongest-effect kernel dataset, 0.81 for the weakest effect, and 0.89 for the ensemble mean) as k in (S3) would yield almost identical results.

Supplementary Methods 4: Radiative forcing from carbon uptake. To compare which of the often opposing effects of a land use change is stronger, or to compute its net effect, the effects of carbon uptake and albedo need to be converted to a common unit. A comprehensive recent review [17] assessed existing strategies and obstacles from original research over the past two decades. We converted cumulative CO_2 uptake or loss to radiative forcing following the simple scheme by Myhre, et al. [18] (Table 3 therein) assuming an instant effect of the airborne fraction, and a linearized radiative forcing (RF) response to concentration change as also described e.g. in Ney, et al. [19] (Eqn. 5-6 therein), but using updated values for the airborne fraction (0.44 [20]) and CO_2 concentration (420 ppm).

Supplementary Methods 5: Uncertainty treatment and combination of CO_2 and α_s based effects. Radiative effects of both, CO_2 uptake and albedo, are expressed as differences in top-of-atmosphere net radiation compared to a "business-as-usual" scenario where the albedo of each site remains unchanged, and its NEP remains unchanged except for the carbon saturation corrections described below.

Especially the CO_2 based radiative forcing of the scenario analysis in the main manuscript (Fig. 2) is subject to a number of known unknowns, i.e. systematic uncertainty. The analysis does not aim at detailed predictions, but at revealing the main differences between land use and management changes aiming to increase NEP, albedo, or both. Even so, it is important to test whether these main differences are robust against variations in assumed unknown variables. We therefore conducted the scenario analysis for each site, year and scenario, for different ensemble members. Each of the following assumed variables were fed into the analysis in two versions, one representing a plausible strong and one a plausible weak effect of land-use change:

Time to fully establish an increased NEP: Whereas changes to land use yielding less net CO_2 uptake (e.g. forest to grassland transition) can typically be finished in one or few years, the opposite (e.g. afforestation) will only yield an NEP typical of the new land-use after years or decades. Based on NEP-age relations of 126 FLUXNET forest sites, Besnard, et al. [21] suggest NEP reaching its final value on average after approximately 20 years, with the

maximum uptake in the total dataset occurring after approximately 30 years. For each change leading to an NEP higher than the site's original one, we therefore assumed a linear transition between the old and new value over 30 years in addition to instantaneous change (0 years).

Time to fully establish the albedo associated with an increased NEP: The albedo of such an ecosystem might also develop over time, albeit possibly faster than NEP. We assumed a linear albedo transition period for the above-mentioned site-wise changes of 0 and 1 times the NEP transition period, respectively.

NBP *vs.* NEP: Carbon exports from a site neither monitored by eddy-covariance systems nor included in the definition of NEP, such as harvested biomass, are relevant for its net atmospheric CO_2 imprint, especially if they respire into CO_2 soon after removal. This is particularly true for crop and grassland ecosystems, where harvested biomass is used as food or fodder typically within few years. The productivity after accounting for such lateral fluxes is termed net biome productivity (NBP) and typically smaller than NEP, unless carbon imports prevail over exports. To frame the effect of an NBP-based CO_2 radiative forcing, we used two ensemble members: In a maximum net sequestration member, RF is based on NEP for all sites except for crop sites, where removal always happens and we used the lowest published removal estimate to our knowledge, 247 gC m^{-2} yr^{-1} [22]. In the minimum net sequestration member, we assumed the highest published removal estimate to our knowledge, 335 gC m^{-2} yr^{-1} [23] for crop sites. Note that both estimates correspond to a (small and considerable, respectively) net mean C loss of cropland. For grassland the maximum removal was set to the mean NEP of this group, 48 gC m^{-2} yr^{-1}, based on a modelling study by Chang, et al. [24] concluding that European (and thus mostly intensively used) grasslands are on average approximately CO_2 neutral. For all other sites, we assumed a maximum removal of 61 gC m^{-2} yr^{-1} as derived from European forest inventories by Luyssaert, et al. [25].

Carbon saturation: Little consensus exists about the net carbon uptake and net biome productivity of aged ecosystems. The abovementioned study consulted for NEP establishment times [21] included forest ages of up to 300 years and showed both, raw data suggesting a possible slight decrease of NEP with forest age from 30 years onwards, and a fitted function of NEP vs. age suggesting approximately constant NEP once the final value has been reached after approximately 30 years. While wetlands can theoretically accumulate unlimited amounts of carbon in growing layers of oxygen-deprived peat, it appears to be expectable that most other ecosystems including forests are more likely to lose CO_2 via respiration proportionally to the amount of soil and biomass carbon already stored, until eventually GPP and R_{eco} (or disturbance-related losses of carbon) cancel out and a maximum storage capacity has been reached. Deriving a rough estimate of maximum aboveground vegetation carbon storage of 25000 gC m^{-2} based on Santoro, et al. [26] assuming a dry biomass C content around 50% [27], and a maximum soil organic carbon storage of 75000 gC m^{-2} based on FAO and ITPS [28], we use the sum (100 000 gC m^{-2}) and 50 % of it (50 000 gC m^{-2}) to frame the uncertainty of future NBP limited by already accumulated C. The difference Δ between this maximum value and cumulative NBP over the synthetic experiment is used to compute a reduction factor to the NBP of each year:

$$\mathrm{NBP} = \mathrm{NBP_{pot}}\left(1 - \frac{\mathrm{NBP_{pot}}}{\Delta - \mathrm{NBP_{pot}}}\right), \quad \text{(S4)}$$

where NBP_{pot} is the biome productivity before considering any saturation effects. The factor asymptotically becomes 1 for large Δ and 0 for Δ approaching 0, such that NBP remains virtually unchanged if accumulated C is far from the maximum, but reduced upon approaching the maximum, which is never exceeded. It is applied whenever NBP is positive. For negative NBP, the same equation (using the absolute value of NBP) is used to limit the carbon loss of sites approaching zero remaining carbon. In this case, Δ is the remaining carbon amount. For lack of more detailed data, each site starts with an organic C stock that is the land surface average of global vegetation and soil carbon stocks according to Friedlingstein, et al. [29], i.e. (450 + 1700) 10^{15} gC (1.483 10^{14} $m^2)^{-1}$.

Instantaneous top-of-atmosphere shortwave radiative effect of albedo changes: We used the model-based albedo kernel datasets predicting the strongest (S18 [11]) and weakest (B19 [13]) effect based on the comparison presented in Supplementary Methods 3. The net effect of albedo changes after atmospheric adjustments and across all wavelengths is subject to further, large uncertainties, which we discuss (Supplementary Methods 3 and main article) based on the current state of knowledge.

Albedo and CO_2-based radiation changes are added for each site, scenario, year and ensemble member separately, and finally averaged across sites. The resulting global time series of net radiation changes per scenario, year and ensemble member are presented in the following Supplementary Figures 5, and the mean, maximum and minimum for each year resulting from the ensemble are used to indicate uncertainty in Fig. 2 of the main article.

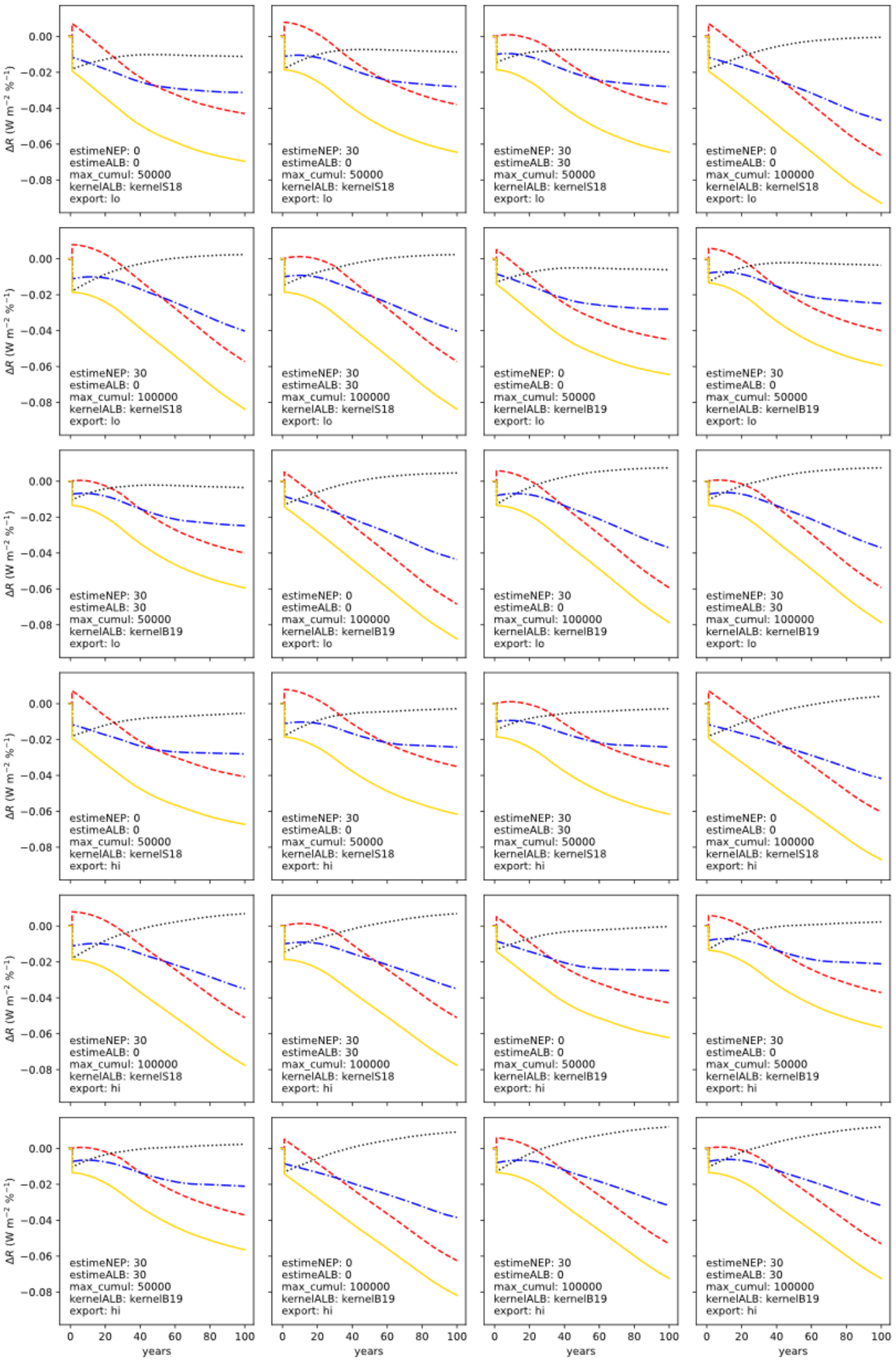

Supplementary Figure 5: Ensemble members underlying the uncertainty bands in Fig. 2 of main text. Scenario 1 (NEP maximisation): red dashed, scenario 2 (albedo maximisation): black dotted, scenario 3 (balanced): blue dash-dotted, scenario 4 (breakthrough): golden solid. Parameters: estimeNEP: Duration (years) of linear ramp-up to an ecosystem with larger NEP. estimeALB: Duration (years) of linear ramp-up of Albedo when changing to an ecosystem with larger NEP. Max_cumul: Amount (gC m^{-2}) of carbon stored in soil and biomass never exceeded. kernelALB: surface albedo kernel dataset used to derive TOA radiation changes from albedo changes. export: land-use type dependent minimum (lo, 0 for all but crop sites) or maximum (hi) C export.

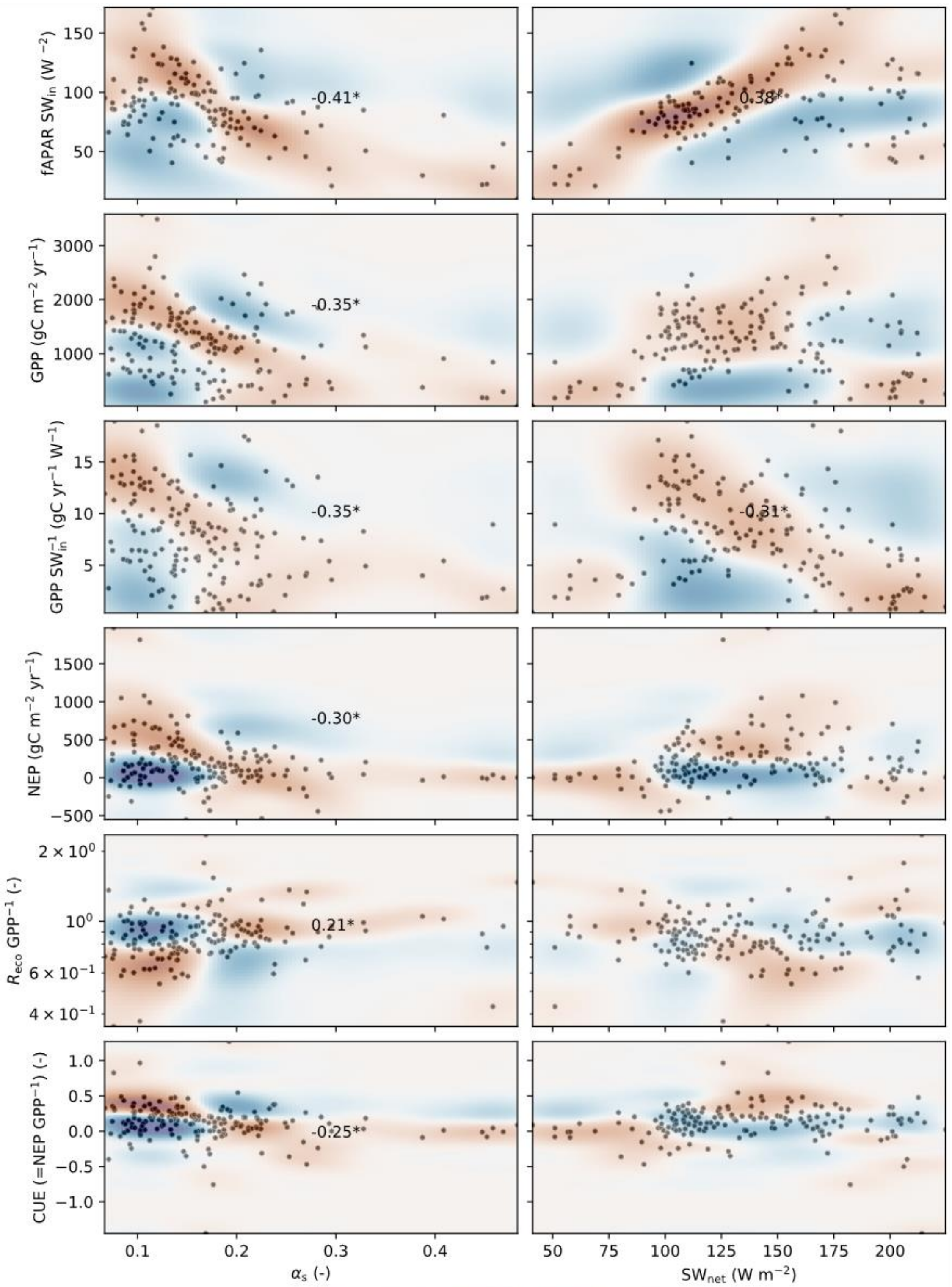

Supplementary Figure 6: Scatterplot as Fig. 3 of main manuscript for more y-axis variables (CUE: carbon use efficiency) and $SW_{net} = SW_{i}n - SW_{out}$ in addition to albedo as x-axis variable. Inset numbers are Spearman correlation coefficients (where significant at p=0.05). Units of color bar (difference between bivariate kernel density and product of univariate kernel densities) are the inverse of the units of the respective Y axis variable.

Supplementary References

1. The matplotlib development team & Whitaker, J. *Basemap Matplotlib Toolkit*, <https://matplotlib.org/basemap/> (2016).
2. Pastorello, G. *et al.* The FLUXNET2015 dataset and the ONEFlux processing pipeline for eddy covariance data. *Scientific Data* **7**, 225 (2020). https://doi.org/10.1038/s41597-020-0534-3
3. Whittaker, R. H. *Communities and Ecosystems*. 2nd edn, (Macmillan, 1975).
4. Kunstler, G. <https://github.com/kunstler/BIOMEplot/blob/master/inst/extdata/biomes.csv> (2014).
5. <https://github.com/zshigrit/python_functions/blob/master/whittaker_biome.py> (2021).
6. Fischer, M. *et al.* Evapotranspiration of a high-density poplar stand in comparison with a reference grass cover in the Czech-Moravian Highlands. *Agric. For. Meteorol.* **181**, 43-60 (2013). https://doi.org/10.1016/j.agrformet.2013.07.004
7. Graf, A. *et al.* Spatiotemporal relations between water budget components and soil water content in a forested tributary catchment. *Water Resources Research* **50**, 4837-4857 (2014). https://doi.org/10.1002/2013wr014516
8. Sun, G. *et al.* Energy and water balance of two contrasting loblolly pine plantations on the lower coastal plain of North Carolina, USA. *Forest Ecology and Management* **259**, 1299-1310 (2010). https://doi.org/10.1016/j.foreco.2009.09.016
9. Sherwood, S. C. *et al.* Adjustments in the forcing-feedback framework for understanding climate change. *Bulletin of the American Meteorological Society* **96**, 217-228 (2015). https://doi.org/10.1175/bams-d-13-00167.1
10. Forster, P. M. *et al.* Recommendations for diagnosing effective radiative forcing from climate models for CMIP6. *Journal of Geophysical Research-Atmospheres* **121**, 12460-12475 (2016). https://doi.org/10.1002/2016jd025320
11. Smith, C. J. *et al.* Understanding Rapid Adjustments to Diverse Forcing Agents. *Geophys. Res. Lett.* **45**, 12023-12031 (2018). https://doi.org/10.1029/2018gl079826
12. Zhao, K. G. & Jackson, R. B. Biophysical forcings of land-use changes from potential forestry activities in North America. *Ecological Monographs* **84**, 329-353 (2014). https://doi.org/10.1890/12-1705.1
13. Bright, R. M. & O'Halloran, T. L. Developing a monthly radiative kernel for surface albedo change from satellite climatologies of Earth's shortwave radiation budget: CACK v1.0. *Geosci. Model Dev.* **12**, 3975-3990 (2019). https://doi.org/10.5194/gmd-12-3975-2019
14. Smith, C. J. *et al.* Effective radiative forcing and adjustments in CMIP6 models. *Atmos. Chem. Phys.* **20**, 9591-9618 (2020). https://doi.org/10.5194/acp-20-9591-2020
15. Pendergrass, A. G., Conley, A. & Vitt, F. M. Surface and top-of-atmosphere radiative feedback kernels for CESM-CAM5. *Earth System Science Data* **10**, 317-324 (2018). https://doi.org/10.5194/essd-10-317-2018
16. Webster, R. Regression and functional relations. *European Journal of Soil Science* **48**, 557-566 (1997). https://doi.org/10.1111/j.1365-2389.1997.tb00222.x
17. Bright, R. M. & Lund, M. T. CO2-equivalence metrics for surface albedo change based on the radiative forcing concept: a critical review. *Atmos. Chem. Phys.* **21**, 9887-9907 (2021). https://doi.org/10.5194/acp-21-9887-2021
18. Myhre, G., Highwood, E. J., Shine, K. P. & Stordal, F. New estimates of radiative forcing due to well mixed greenhouse gases. *Geophys. Res. Lett.* **25**, 2715-2718 (1998). https://doi.org/10.1029/98gl01908
19. Ney, P. *et al.* CO2 fluxes before and after partial deforestation of a Central European spruce forest. *Agric. For. Meteorol.* **274**, 61-74 (2019). https://doi.org/10.1016/j.agrformet.2019.04.009
20. IPCC. *Climate Change 2021: The Physical Science Basis. Contribution of Working Group I to the Sixth Assessment Report of the Intergovernmental Panel on Climate Change*. (Cambridge University Press, 2021).
21. Besnard, S. *et al.* Quantifying the effect of forest age in annual net forest carbon balance. *Environ. Res. Lett.* **13** (2018). https://doi.org/10.1088/1748-9326/aaeaeb
22. Ciais, P. *et al.* The European carbon balance. Part 2: croplands. *Global Change Biology* **16**, 1409-1428 (2010). https://doi.org/10.1111/j.1365-2486.2009.02055.x
23. Kutsch, W. L. *et al.* The net biome production of full crop rotations in Europe. *Agriculture Ecosystems & Environment* **139**, 336-345 (2010). https://doi.org/10.1016/j.agee.2010.07.016
24. Chang, J. F. *et al.* The greenhouse gas balance of European grasslands. *Global Change Biology* **21**, 3748-3761 (2015). https://doi.org/10.1111/gcb.12998
25. Luyssaert, S. *et al.* The European carbon balance. Part 3: forests. *Global Change Biology* **16**, 1429-1450 (2010). https://doi.org/10.1111/j.1365-2486.2009.02056.x
26. Santoro, M. *et al.* The global forest above-ground biomass pool for 2010 estimated from high-resolution satellite observations. *Earth System Science Data* **13**, 3927-3950 (2021). https://doi.org/10.5194/essd-13-3927-2021
27. Dewar, R. C. & Cannell, M. G. R. Carbon sequestration in the trees, products and soils of forest plantations - an analysis using UK examples. *Tree Physiology* **11**, 49-71 (1992). https://doi.org/10.1093/treephys/11.1.49
28. FAO & ITPS. Global Soil Organic Carbon Map V1.5: Technical report., (Rome, 2020).
29. Friedlingstein, P. *et al.* Global Carbon Budget 2021. *Earth System Science Data* **14**, 1917-2005 (2022). https://doi.org/10.5194/essd-14-1917-2022
